# *Mentha piperita* Essential Oil in Olive Oil: Extending Erythrocyte Viability and Limiting Bacterial Growth Under Serum-Free Conditions

**DOI:** 10.3390/molecules31030516

**Published:** 2026-02-02

**Authors:** Tina Novaković, Emina Mehmedović, Maja Krstić Ristivojević, Ivana Prodić, Vesna Jovanović, Milica Aćimović, Katarina Smiljanić

**Affiliations:** 1 University Clinical Centre of Serbia, University of Belgrade—Faculty of Medicine, 11000 Belgrade, Serbia; tinkanova@gmail.com; 2 Faculty of Technology and Faculty of Natural Sciences and Mathematics, University of Tuzla, 75000 Tuzla, Bosnia and Herzegovina; emiina.mehmedovic@gmail.com; 3 CoE for Molecular Food Sciences, Department of Biochemistry, University of Belgrade—Faculty of Chemistry, Studentski trg 12-16, 11158 Belgrade, Serbia; krstic_maja@chem.bg.ac.rs (M.K.R.); vjovanovic@chem.bg.ac.rs (V.J.); 4 Institute of Virology, Vaccines and Sera “Torlak”—National Institute of the Republic of Serbia, Vojvode Stepe 458, 11152 Belgrade, Serbia; iprodic@torlak.rs; 5 Institute of Field and Vegetable Crops—National Institute of the Republic of Serbia, 21101 Novi Sad, Serbia; milica.acimovic@ifvcns.ns.ac.rs

**Keywords:** ATP-oil^®^, bacterial contamination, erythrocyte survival, peppermint essential oil, red blood cell, serum-free culture, transfusion safety

## Abstract

Background: Serum-free culture of red blood cells (RBCs) typically leads to rapid loss of viability, limiting experimental and translational applications. Lipid-rich formulations and essential oils may provide biocompatible support for RBC integrity while limiting microbial overgrowth. Methods: RBCs from nine healthy adult donors were cultured in serum-free RPMI under four conditions: control, vehicle (olive oil, 1:100 *v*/*v*), genuine adenosine triphosphate (ATP)-oil^®^ (1:100 *v*/*v*), and laboratory oil, “mimicking” ATP-oil^®^. Cultures were maintained for 18 days. Viability was assessed by light microscopy and trypan blue exclusion; bacterial contamination was qualitatively observed on day 18. Results: Genuine ATP-oil^®^ maintained 35–45% RBC viability at day 18, whereas control and vehicle cultures declined rapidly. The mimicking preparation did not reproduce these effects. ATP-oil^®^ immersion was associated with a qualitative reduction in bacterial contamination versus control, consistent with a dual action on RBC preservation and microbial suppression under serum-free conditions. Conclusions: Supplementation with ATP-oil^®^ substantially prolongs RBC survival and limits bacterial overgrowth in vitro, outperforming commonly used serum or plasma supplements on a per-volume basis. These findings suggest potential applications for improving ex vivo handling or storage of blood components and for reducing background contamination in diagnostic microbiology. Further studies with larger cohorts are warranted to reveal underlying mechanisms and to define active constituents in order to standardize production.

## 1. Introduction

The survival of red blood cells (RBCs) in vitro has historically been examined primarily in the context of transfusion medicine, with emphasis on storage, compatibility, and post-transfusion recovery [1]. More recently, RBCs have been recognized not only as passive oxygen carriers but also as modulators of immune responses, intercellular communication, and tissue homeostasis, prompting renewed interest in their biology under culture conditions [2]. However, in serum-free RPMI media, RBC longevity remains limited: up to ~50% of purified RBCs may persist to day 6, whereas by day 10, only a small fraction typically remains viable [3,4]. Attempts to prolong survival using serum or plasma supplements can extend culture duration, but often at the expense of viability and functional integrity [3,4].

Adenosine triphosphate (ATP)-oil^®^ is an α-τ© preparation composed of virgin olive oil and essential oil of *Mentha piperita* L., intended primarily for gastrointestinal and dermatological use. According to product specifications, it is proposed to support detoxification and energy balance, although these claims are insufficiently substantiated by controlled studies. The individual components are biologically plausible in this context: polyphenols and monounsaturated fatty acids in virgin olive oil exhibit antioxidant and membrane-protective effects that can mitigate oxidative damage and lipid peroxidation [5], whereas peppermint essential oil has been reported to exert antibacterial activity against a broad spectrum of Gram-positive and Gram-negative bacteria [6]. Together, these attributes suggest a testable rationale for improving RBC stability while limiting microbial overgrowth in serum-free culture.

We set out to evaluate whether ATP-oil^®^ supplementation could (i) prolong RBC survival in a serum-free culture model and (ii) reduce bacterial contamination, using RBCs from healthy adult donors under well-controlled, side-by-side treatment conditions.

## 2. Results

### 2.1. Patients and Compliance with the Detoxification Regimen

Compliance results were assessed using a structured questionnaire and are presented together with demographic data and baseline RBC counts (Table 1).

### 2.2. Red Blood Cells Survival in Serum-Free Culture

**Figure 1:**
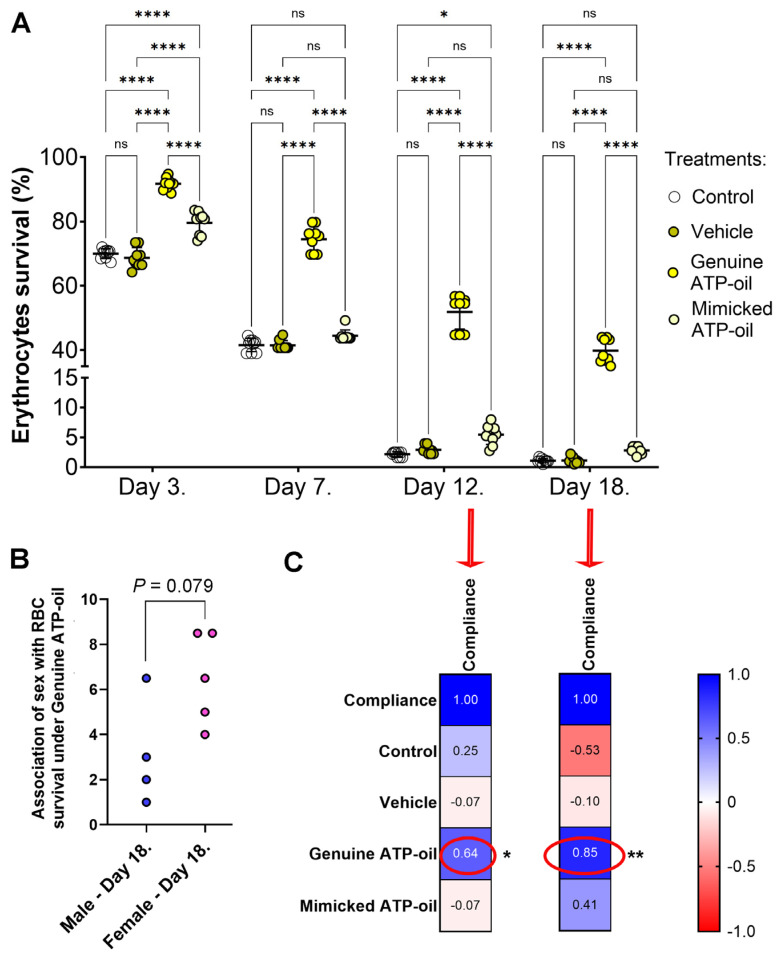
Effects of olive oil infused with essential oil-based treatments on red blood cell (RBC) survival and donor compliance under serum-free conditions. RBC survival under four culture conditions across four time points was analyzed by two-way ANOVA with post hoc tests (**A**); the association between sex and RBC survival under genuine ATP-oil^®^ on day 18 was analyzed by the Mann–Whitney test (**B**); and the correlation between donor compliance and RBC survival under genuine ATP-oil^®^ was analyzed by Spearman correlation (**C**). Statistical annotations: **** *p* < 0.0001, ** *p* < 0.01, * *p* < 0.05, ns not significant. Abbreviation: RBC, red blood cell.

Across the nine donors, RBCs cultured in serum-free RPMI showed comparable survival profiles under four conditions (Figure S1). In cultures supplemented with genuine ATP-oil^®^ (1:100 *v*/*v*), viability remained within 35–45% on day 18 (Figure 1A). In contrast, control and vehicle (olive oil, 1:100 *v*/*v*) conditions declined rapidly over time, approaching minimal viability by days 12–18 (Figure 1A).

#### 2.2.1. Genuine ATP-Oil^®^ Versus Vehicle and Mimicking Preparation

The vehicle did not improve survival relative to control at any time point (Figure 1A). The laboratory “mimicking” ATP-oil (same component ratios; 0.7% *v*/*v* EO) did not reproduce the effect of the genuine product: day-18 viability in mimicking wells was substantially lower than with genuine ATP-oil^®^ and comparable to vehicle (Figure 1A).

#### 2.2.2. Donor-to-Donor Consistency and Compliance

Between-donor variability in the shape of RBC survival curves was low-to-modest (Figure S1). All donors demonstrated prolonged survival under genuine ATP-oil^®^ (Figure 1A). Interestingly, no associations between sex and the four applied conditions were observed, except under genuine ATP-oil^®^, which, although not statistically significant, showed a trend toward higher RBC survival in females (Figure 1B). This trend could likely become a statistically significant association if evaluated in a larger study cohort. Day-18 viability in the ATP-oil^®^ condition tracked with donor compliance to the two-week ingestion regimen (Table 1), with higher compliance generally corresponding to greater RBC survival in vitro (Figure 1C). This was especially evident on days 12/18, as correlation analysis revealed a moderate positive significant association on day 12 and a strong positive significant association on day 18.

### 2.3. Reduction in Bacterial Contamination

**Figure 2:**
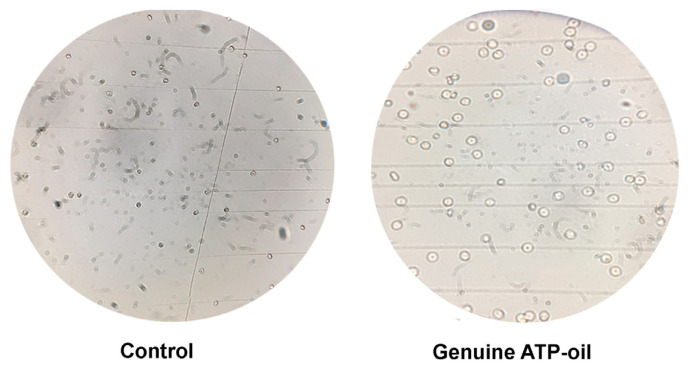
Representative bright-field microscopy of RBC cultures on day 18: control vs. genuine ATP-oil^®^ (1:100 *v*/*v*). Images acquired at 2000× total magnification.

Microscopic assessment on day 18 revealed a marked qualitative reduction in bacterial contamination in cultures supplemented with genuine ATP-oil^®^ compared with controls (Figure 2). Representative images show predominantly intact RBCs with only discrete bacterial forms in ATP-oil^®^-treated wells, versus dense bacterial overgrowth and lysed cell membranes in controls.

## 3. Discussion

### 3.1. Interpretation of Findings

All subjects exhibited comparable profiles of RBC survival under the four tested conditions. The most striking observation was the markedly prolonged survival of RBCs in the genuine ATP-oil^®^ group, which consistently maintained 35–50% viability at day 18. By contrast, RBCs in control and vehicle conditions declined rapidly, in agreement with previous studies showing that erythrocytes rarely persist beyond 10 days in serum-free RPMI medium [3,4]. Even when serum or plasma supplements are used, viability rarely exceeds 2% by day 18, underscoring the exceptional effect of ATP-oil^®^ at only 1% supplementation compared with conventional 10% serum additions [4].

### 3.2. Mechanistic Considerations

These findings suggest that ATP-oil^®^ stabilizes RBC membranes under serum-free conditions. Virgin olive oil-derived polyphenols and monounsaturated fatty acids are known to delay eryptosis, reduce oxidative stress, and protect against lipid peroxidation [7,8,9], thereby preserving cell membrane integrity. In parallel, peppermint essential oil, characterized in our previous study [10], is rich in menthol, menthone, and 1,8-cineol. It exhibits antibacterial activity against both Gram-positive and Gram-negative bacteria via disruption of bacterial membranes, altered permeability, and enzyme inhibition [11,12]. The combined antioxidant and antimicrobial actions likely underlie the qualitative reduction in bacterial contamination observed in ATP-oil^®^-treated cultures (Figure 2). Indeed, intact RBCs with only discrete bacterial presence were consistently observed with ATP-oil^®^, whereas control cultures showed extensive bacterial growth and lysis.

### 3.3. Implications in Transfusion Medicine and Diagnostics

Maintenance of sterility is a critical parameter in both ex vivo storage and transfusion safety [13,14]. Bacterial contamination remains a leading cause of adverse transfusion reactions, despite donor screening and testing [14]. The ability of ATP-oil^®^ to suppress microbial proliferation suggests potential as an adjunct to improve microbiological safety of stored blood products. Beyond transfusion medicine, this effect may be relevant to microbiological diagnostics. Urine cultures, among the most frequently performed microbiological tests, are highly susceptible to contamination, often resulting in false-positive results and inappropriate antimicrobial therapy [15]. Olive oil polyphenols interfere with bacterial adhesion and biofilm formation [16], while peppermint essential oil exhibits broad-spectrum antibacterial activity [6]. Although speculative at this stage, ATP-oil^®^ or its bioactive components could improve diagnostic reliability by limiting background contamination in biological samples such as urine.

### 3.4. Limitations and Future Directions

An additional point of interest was the discrepancy between genuine and mimicking ATP-oil^®^ preparations, despite identical composition and concentration of essential oil. This may reflect subtle variations in preparation, oxidative status, or contaminants introduced during handling, underscoring the importance of standardization and quality control. Furthermore, the positive correlation between donor compliance with ATP-oil^®^ ingestion and RBC survival suggests that prior systemic exposure may prime RBC membranes through lipid incorporation. However, the study is limited by its small sample size (n = 9), qualitative assessment of bacterial contamination, and absence of mechanistic assays. Another limitation of this study is that neither serum nor whole blood samples were inoculated on nutrient media to directly quantify bacterial colony-forming units (CFU/mL). Such measurements would have provided stronger evidence for reduced bacterial growth under α-tau treatment, but could not be retrospectively applied since fresh blood from the same donors is no longer available. Future clinical studies will therefore include CFU/mL determinations to provide deeper mechanistic insights. As bacterial reduction in this study was assessed qualitatively by microscopy, upcoming work should also incorporate quantitative assays (e.g., CFU counts or qPCR) to validate these observations. In addition, further research should include proteomic and lipidomic profiling, ATP quantification, standardized microbiological assays, and expanded donor cohorts to validate and extend these preliminary findings.

## 4. Materials and Methods

### 4.1. Study Cohort and Ethics Approval

Nine adult volunteers, clinically evaluated as healthy and without hematological or chronic systemic disorders, were enrolled at Beo-Lab Clinic (Belgrade, Serbia) for blood collection and complete blood cell count analysis. Blood was drawn into citrate-containing Vacutainer^®^ tubes (Becton-Dickinson, Franklin Lakes, NJ, USA). Two weeks before blood donation, participants followed a detoxification program consisting of daily ingestion of ATP-oil^®^ (50 mL/day).

The study was conducted in accordance with the Declaration of Helsinki and approved by the Ethics Committee of the University of Belgrade—Faculty of Chemistry (protocol no. 1-10/22). Written informed consent was obtained from all donors prior to blood collection.

### 4.2. Reagents

RPMI-1640 medium was obtained from PAA—The Cell Culture Company (Linz, Austria). L-glutamine and all other reagents were purchased from Sigma-Aldrich (St. Louis, MO, USA) and were of analytical grade or higher. Deionized water used in all experiments was produced using a Smart2Pure purification system (Barnstead/Thermo Fisher Scientific, Waltham, MA, USA). Peppermint essential oil (*Mentha piperita* aetheroleum; derived from *Mentha piperita* cv. Danica L.), used to prepare the genuine ATP-oil^®^ and the mimicking ATP-oil, was obtained from the Institute of Field and Vegetable Crops (Novi Sad, Serbia) and met pharmacopeia purity requirements.

### 4.3. Mentha Piperita Essential Oil Extraction

*Mentha piperita* cv. ‘Danica’, originating from the Institute of Field and Vegetable Crops Novi Sad (IFVCNS) collection, was verified and deposited in the herbarium of the University of Novi Sad (BUNS). Steam distillation was carried out on pilot-scale stainless steel equipment at IFVCNS under atmospheric pressure, using 100 kg of dried *Mentha piperita* plant material per run. The material was loaded into a lidded cylindrical vessel and exposed to steam, which entrained volatile compounds and directed them into a cooler and condenser. The condensate was collected in a Florentine flask over a 4 h period, in line with European pharmacopeia (2010) requirements of at least 3 h. The essential oil, separated as a layer above the hydrolate, was decanted into a separatory funnel and dried over anhydrous sodium sulfate.

The comprehensive characterization of the *Mentha piperita* cv. ‘Danica’ essential oil used in this study was analyzed in detail in our previous work “Multistep Approach Points to Compounds Responsible for the Biological Activity and Safety of Hydrolates from Nine Lamiaceae Medicinal Plants on Human Skin Fibroblasts” [10]. The chemical composition of the oil corresponds to the menthone–menthol chemotype, characterized by high levels of menthol and menthone as dominant constituents, alongside minor amounts of 1,8-cineol and other monoterpenoids. This chemotypic profile is important to clarify, as different peppermint chemotypes may vary considerably in biological activity and safety.

### 4.4. Preparation of the Mimicking ATP-Oil

The laboratory “mimicking” ATP-oil was prepared using the same component ratios as the genuine product, with peppermint essential oil adjusted to 0.7% (*v*/*v*) by volumetric addition. The mixture was homogenized under sterile conditions, protected from light, stored at 4 °C, and used within 24 h of preparation.

### 4.5. Cell Culture Design

Whole blood aliquots were initially diluted ~100-fold in RPMI-1640 medium to achieve a uniform intermediate concentration of 0.05 × 10^9^ RBCs/mL for all participants. Following a further 10-fold dilution, 5 × 10^6^ RBCs/mL were seeded per well in 96-well culture plates (Sarstedt, Newton, NC, USA). Cultures were maintained at 37 °C in a humidified atmosphere with 6% CO_2_.

Four experimental conditions were established:

- Control: RPMI-1640 supplemented with 5 mM glucose and 1 mM glutamine.
- Vehicle: Control medium with olive oil (1:100 *v*/*v*).
- Genuine ATP-oil^®^: Vehicle medium supplemented with ATP-oil^®^ (1:100 *v*/*v*).
- Mimicking ATP-oil: Vehicle medium supplemented with laboratory-prepared ATP-oil (1:100 *v*/*v*).

Both genuine ATP-oil^®^ and mimicking ATP-oil preparations contained identical base components, with the essential oil fixed at 0.7% (*v*/*v*). The only difference was the personnel and procedure used in preparation.

### 4.6. Microscopy and Cell Counting

Visualization and enumeration of RBCs were performed using a Neubauer hemocytometer (Paul Marienfeld GmbH & Co. KG, Lauda-Königshofen, Germany) with trypan blue exclusion, with minor modifications as described herein. Briefly, 10 μL of suspension from each well was loaded into the counting chamber and examined by light microscopy (Motic, Kowloon, Hong Kong SAR, China) at 400× total magnification. Images were acquired using a Canon IXUS 400 camera (Canon Inc., Tokyo, Japan) coupled to the microscope (additional ~5× optical zoom) for documentation.

Red blood cell viability was assessed by trypan blue exclusion at days 3, 7, 12, and 18. A 0.2% trypan blue solution was mixed with five-fold concentrated phosphate-buffered saline (PBS) at a 4:1 *v*/*v* ratio, followed by dilution with the culture suspension (1:1 *v*/*v*). The percentage of viable cells was calculated as follows:

(1) $$\mathrm{Viability}\left(\%\right)=\frac{\mathrm{Number}\;\mathrm{of}\;\mathrm{viable}\;\mathrm{cells}\times 100}{\mathrm{Number}\;\mathrm{of}\;\mathrm{viable}\;\mathrm{cells}+\mathrm{Number}\;\mathrm{of}\;\mathrm{dead}\;\mathrm{cells}}.$$

where viable cells exclude trypan blue uptake, and dead cells include trypan blue–positive counts.

For each donor and condition at each time point, two wells were plated (duplicates). Within each well, two independent 20 µL subsamples were loaded and assessed by trypan blue exclusion (technical duplicates), yielding four replicate measurements in total.

### 4.7. Statistical Analysis

For each donor, technical replicates (two wells × two counts per well) were averaged to yield a single donor-level value per condition and time point; the donor was treated as the experimental unit (n = 9). Differences among treatments across time were assessed with a two-way repeated-measures ANOVA (factors: treatment and time), followed by Tukey’s post hoc test for multiple comparisons. For assessment of associations between sex and the applied conditions, the non-parametric Mann–Whitney U test was used, while Spearman’s rank correlation was applied to evaluate trends between donor compliance and treatments. The significance threshold was set at α = 0.05. Analyses were performed in GraphPad Prism version 10.5 (GraphPad Software, San Diego, CA, USA). Symbols in figures denote significance levels: *p* < 0.05 (*), *p* < 0.01 (**), *p* < 0.0001 (***); Tukey-adjusted for multiple comparisons. The statistical dataset, in the form of a GraphPad Prism file that supports the findings of this study, is openly available in the Zenodo repository [17]. Assessment of bacterial contamination was qualitative (microscopy-based) and was not subjected to formal hypothesis testing.

## 5. Conclusions

This study demonstrates that supplementation with genuine ATP-oil^®^ can prolong red blood cell survival under serum-free conditions while simultaneously reducing bacterial contamination. These findings point to ATP-oil^®^ as a promising biocompatible strategy for improving blood product storage and minimizing background contamination in microbiological applications. Future clinical studies with larger cohorts and mechanistic analyses are warranted to validate these results, support standardized production, and explore opportunities for patent development related to nutraceutical formulations designed to enhance erythrocyte survival and suppress microbial growth.

## Figures and Tables

**Table 1:** Patient cohort characteristics, baseline RBC counts, and α-τ© regimen compliance scores expressed as percentages.

ID	Age	Sex	RBC Count (×10^12^/L)	Compliance to Detoxification Regimen	Cell Count Parameters Out of Range (≥7% of Border Limit)
1.	41	M	5.21	75–100%	-
2.	49	F	4.49	75–100%	-
3.	50	F	4.31	75–100%	-
4.	41	F	4.61	75–100%	↓ Neutrophils 1500/µL
5.	66	M	4.97	50–75%	-
6.	61	F	4.03	50–75%	↓ Neutrophils 1800/µL
7.	41	M	4.63	25–50%	-
8.	43	M	5.10	25–50%	-
9.	61	F	4.64	25–50%	-

Compliance (%) indicates self-reported adherence to the 14-day ATP-oil^®^ ingestion protocol (50 mL/day). “Cell count parameters out of range” refers to hematological values falling ≥7% below the lower reference limit; ↓ indicates a decreased value relative to the normal range.

## Data Availability

Data supporting the findings of this study, related to Figure 1 and Figure S1, are openly available in the Zenodo repository at https://doi.org/10.5281/zenodo.18133073 with dataset title “Research data—ATP-oil effects on erythrocytes survival in culture statistics for Figure 1.”

## Supplementary Materials

The following supporting information can be downloaded at: https://www.mdpi.com/article/10.3390/molecules31030516/s1. Figure S1: Genuine ATP-oil^®^ (1:100 *v*/*v*) sustains erythrocyte viability over 18 days across nine independent donors marked with serial numbers.

## Abbreviations

The following abbreviations are used in this manuscript:

ATP	adenosine triphosphate
ATP-oil^®^	formulation of virgin olive oil and peppermint essential oil (α-τ©)
CO_2_	carbon dioxide
EO	essential oil
FBS	fetal bovine serum
PBS	phosphate-buffered saline
PPP	platelet-poor plasma
RBC	red blood cells
RPMI	Roswell Park Memorial Institute medium
SD	standard deviation
*v*/*v*	volume per volume
