# Supplementary material for: Mentha piperita Essential Oil in Olive Oil: Extending Erythrocyte Viability and Limiting Bacterial Growth Under Serum-Free Conditions
Source: Molecules. 2026 Feb 2;31(3):516. doi: 10.3390/molecules31030516 (PMC12899880; doi:10.3390/molecules31030516)
Supplement: Supplementary file 1 [file molecules-31-00516-s001.zip › molecules-4050263-supplementary.pdf]

Supplementary Information

Communication

Mentha piperita Essential Oil in Olive Oil: Extending Erythrocyte Viability and Limiting Bacterial Growth Under Serum-Free Conditions

Tina Novaković [1,†], Emina Mehmedović [2,†], Maja Krstić Ristivojević [3], Ivana Prodić [4], Vesna Jovanović [3], Milica Aćimović [5] and Katarina Smiljanić [3,*]

[1] University Clinical Centre of Serbia, University of Belgrade—Faculty of Medicine, 11000 Belgrade, Serbia; tinkanova@gmail.com
[2] Faculty of Technology and Faculty of Natural Sciences and Mathematics, University of Tuzla, Tuzla 75000, Bosnia and Herzegovina; emiina.mehmedovic@gmail.com
[3] CoE for Molecular Food Sciences, Department of Biochemistry, University of Belgrade—Faculty of Chemistry, Studentski trg 12-16, 11158 Belgrade, Serbia; krstic_maja@chem.bg.ac.rs (M.K.R.); vjovanovic@chem.bg.ac.rs (V.J.)
[4] Institute of Virology, Vaccines and Sera "Torlak"—National Institute of the Republic of Serbia, Vojvode Stepe 458, 11152 Belgrade, Serbia; iprodic@torlak.rs
[5] Institute of Field and Vegetable Crops—National Institute of the Republic of Serbia, 21101 Novi Sad, Serbia; milica.acimovic@ifvcns.ns.ac.rs
* Correspondence: katarinas@chem.bg.ac.rs
† These authors contributed equally to this work.

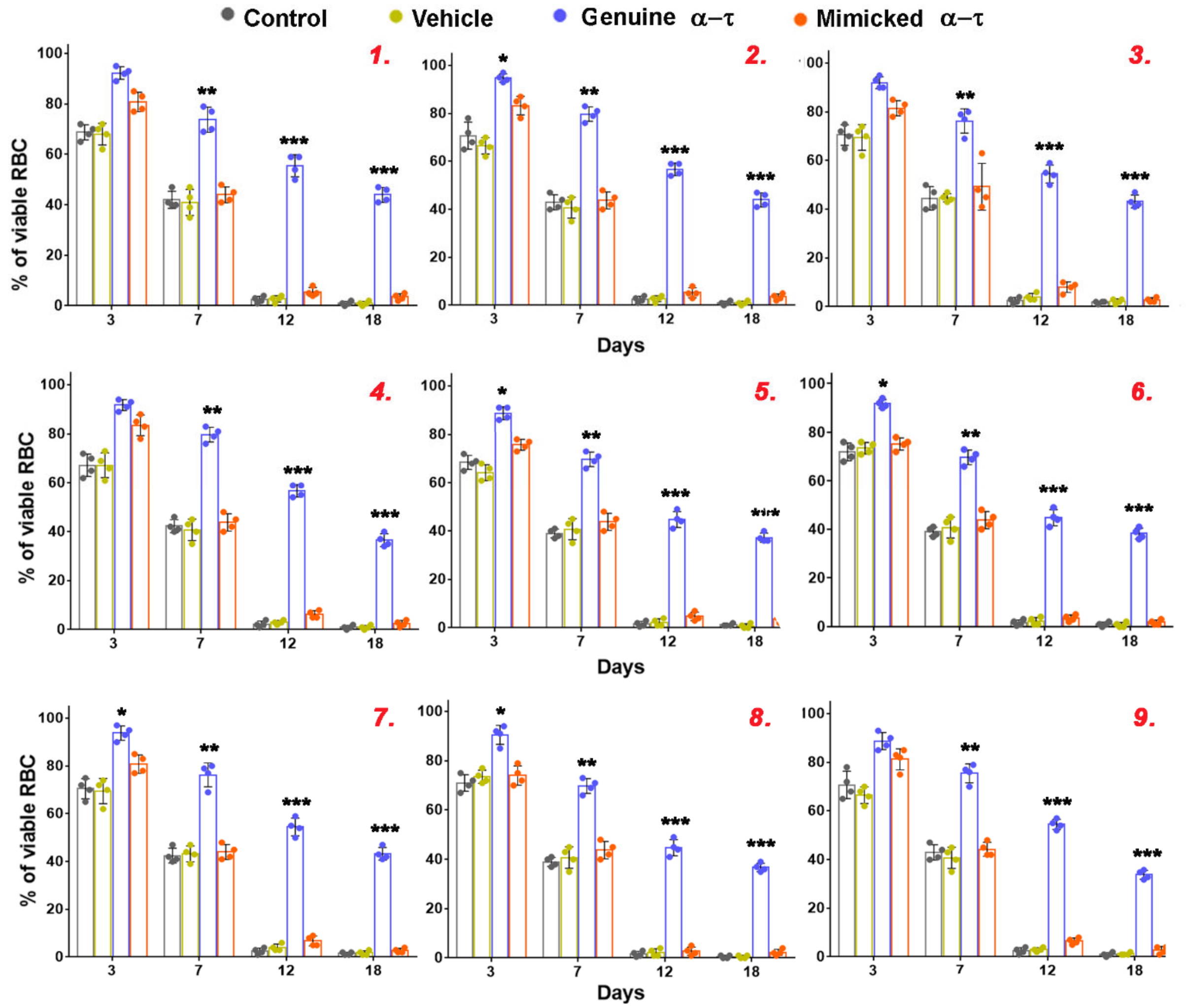

Figure S1. Genuine ATP-oil® (1:100 v/v) sustains erythrocyte viability over 18 days across nine independent donors marked with serial numbers. Percentage of viable red blood cells (RBCs; trypan blue exclusion) at days 3, 7, 12, and 18 under four conditions: Control, Vehicle (olive oil, 1:100 v/v), Genuine ATP-oil® (1:100 v/v), and mimicking ATP-oil (1:100 v/v). Panels 1–9 correspond to independent donors numerated as per Table 1; within each panel, bars show mean ± SD of two wells (each counted in duplicate), and dots indicate individual technical counts. Symbols *, **, and *** indicate Tukey-adjusted significance versus the other treatments at $p < 0.05$, $p < 0.01$, and $p < 0.001$, respectively. Abbreviations: RBC, red blood cells.
